# Supplementary material for: Mitochondria are required for pro‐ageing features of the senescent phenotype
Source: EMBO J. 2016 Feb 4;35(7):724–42. doi: 10.15252/embj.201592862 (PMC4818766; doi:10.15252/embj.201592862)
Supplement: Supplementary file 1 — Appendix [file EMBJ-35-724-s001.pdf]

Correia-Melo *et al*. Appendix

Table of contents:

Appendix Supplementary Methods;

3 Appendix Supplementary Figures;

4 Appendix Supplementary Tables.

Appendix Supplementary methods:

Intraperitoneal Glucose Tolerance Test (IPPGT)

An intraperitoneal injection of glucose (1g/kg body weight) was performed in overnight fasted male mice. Blood glucose levels were monitored from the tail vein with a glucose meter One-Touch Ultra glucose meter (Lifescan, Milpitas, CA) from a 4 µl sample of tail blood at 0, 10, 20, 30, 60 and 90 minutes after glucose injection.

Intraperitoneal insulin Tolerance Test (IPITT)

Mice were injected intraperitoneally with 1 U/Kg of human insulin (Actrapid, Novo Nordisk, Denmark) after 6h of fasting at 14h. Blood glucose levels were measured as aforementioned for the GTT.

Oxygen consumption and Respiratory exchange ratio in PGC-1β$^{-/-}$ animals

Comprehensive laboratory animal monitoring system (Columbus Instruments, Ohio, USA) was run at 21-22°C. Oxygen consumption and Carbon dioxide release were measured by monitoring system (Minimox system built by P. Murgatroyd) over 72 hours after a 72h acclimatising period the week before. Respiratory exchange ratio was obtained from the CO_2/O_2 ratio.

3D Electron Microscopy

Samples were fixed in 2.5% glutaraldehyde, 2% paraformaldehyde in 0.1 M sodium cacodylate buffer pH 7.4, and post-fixed in 2% osmium tetroxide in 0.1 M cacodylate buffer with 0.15% potassium ferrocyanide. After rinsing in buffer, samples were dehydrated through a series of graded ethanol to propylene oxide solutions, infiltrated, embedded in epoxy resin, and polymerized at 70°C overnight. After initial fixation, samples for 3D EM were stained with a modified heavy-metal staining method (Peres et al. 2014) and processed through a graded series of alcohol and propylene oxide solutions and finally embedded in Epon hard resin (Denk and Horstmann 2004). Thick sections of 0.5 microns were cut to determine the correct area to analyze and coated with iridium in a Denton Desk II sputter coater. The 3D EM images were collected with a thickness of 20 nm on a FEI Helios Nanolab 660 Dualbeam system using Auto Slice and View software. The image analysis was performed using FIJI software (Fiji.sc/Fiji) an image-processing package based on ImageJ.

Supplementary references

Denk, W., and Horstmann, H. (2004). Serial block-face scanning electron microscopy to reconstruct three-dimensional tissue nanostructure. PLoS biology *2*, e329.

Perez, A.J., Seyedhosseini, M., Deerinck, T.J., Bushong, E.A., Panda, S., Tasdizen, T., and Ellisman, M.H. (2014). A workflow for the automatic segmentation of organelles in electron microscopy image stacks. Frontiers in neuroanatomy *8*, 126.

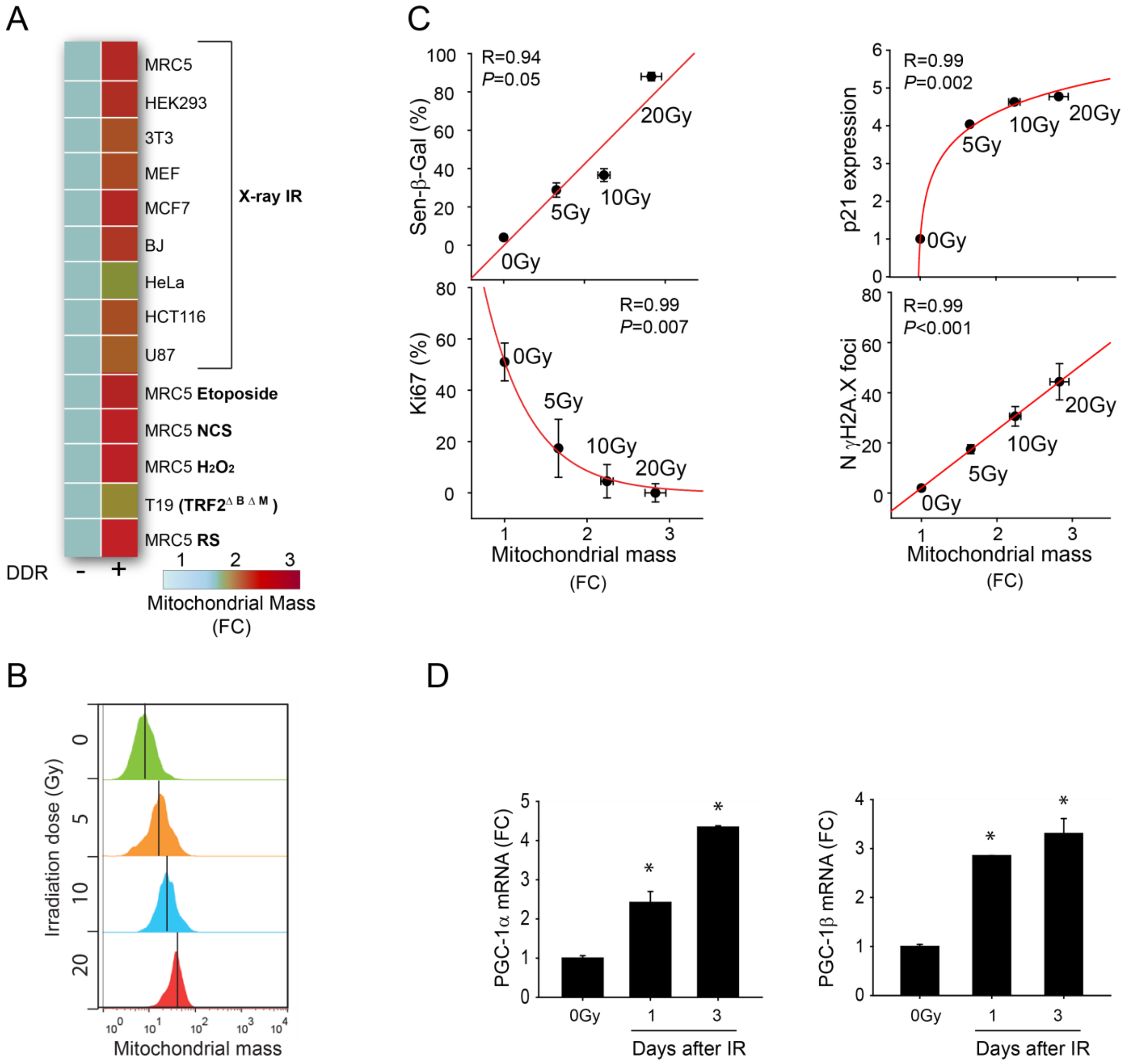

Appendix Figure S1 Mitochondrial content increases as a result of a DDR. A) Mitochondrial mass analysis (NAO intensity) 2-4 days following replication exhaustion (RS), genotoxic stress (X-ray irradiation, Etoposide, Neocarcinostatin (NCS), H_2O_2) or telomere dysfunction ($TRF2^{\Delta B \Delta M}$) in a variety of cell lines. Data are mean from 3 independent experiments per cell line or treatment; **B)** Mitochondrial mass increases proportionally to irradiation dose (Gy X-ray) in MRC5 fibroblasts. Data are representative histograms of NAO fluorescence measured by flow cytometry from 3 independent experiments; **C)** Graphs showing a correlation between mitochondrial mass, Senescence-associated β-Galactosidase (Sen-β-Gal), Ki67 and number of γH2A.X foci (measured by histochemistry or immunofluorescence stainings) and p21 expression (measured by western blotting). Data are mean±S.E.M of n=3 independent experiments. **D)** mRNA expression (FC) of *PGC-1α* and *PGC-1β* in human young proliferating MRC5 fibroblasts (0Gy) and 1 and 3 days after 20Gy X-ray. Expression was normalized to mRNA levels of *GAPDH*. Data are mean±S.E.M of n=3 independent experiments. Asterisks denote statistical significant $P<0.05$ One-way ANOVA.

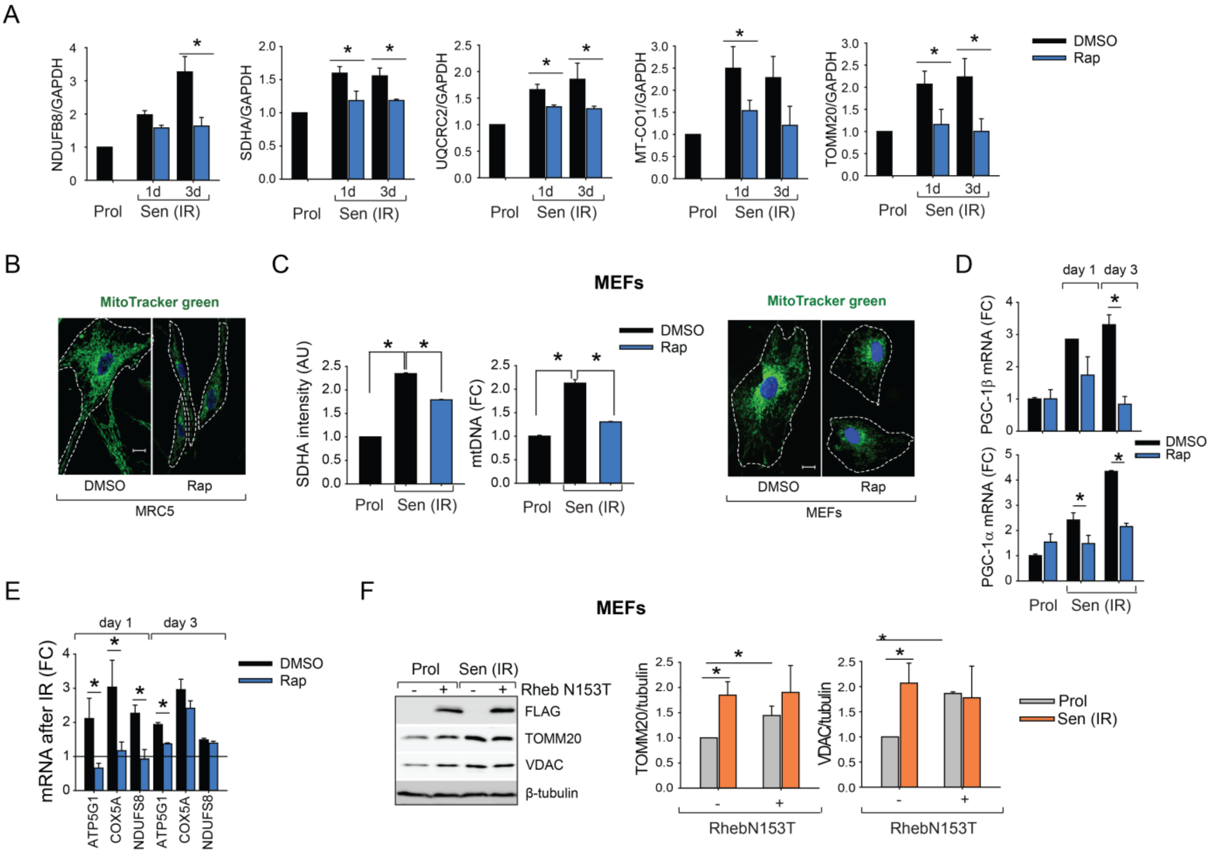

Appendix Figure S2 mTORC1 integrates DDR signals towards mitochondrial biogenesis during cellular senescence. A) Quantification of mitochondrial proteins expression in MRC5 fibroblasts, 1 and 3 days after 20Gy X-ray in the presence or absence of 100nM rapamycin. Data are mean±S.E.M. of n=3 independent experiments; **B)** Representative images of human MRC5 fibroblasts stained with Mito Tracker green 3 days after 20Gy X-ray irradiation with and without 100nM rapamycin treatment. Scale bar=10μm. Data are representative of 3 independent experiments; **C)** (left) Quantification of SDHA, (middle) mtDNA copy number and (right) representative images of Mito Tracker green staining in MEFS treated with or without 100nM rapamycin, 3 days after 10Gy X-ray irradiation. Data are mean±S.E.M of n=3 independent experiments; Scale bar=10μm. Data are representative of 3 independent experiments; **D)** mRNA expression fold change (FC) of *PGC-1α* and *PGC-1β* in proliferating and senescent (3 days after 20Gy X-ray) MRC5 fibroblasts with or without 100nM Rapamycin. Expression was normalized to mRNA levels of *GAPDH*. Data are mean±S.E.M of n=3 independent experiments; **E)** mRNA expression (FC) of the mitochondrial genes *ATP5G1*, *COX5A* and *NDUFS8* after 20Gy X-ray with or without rapamycin treatment in MRC5 fibroblasts. Expression was normalized to mRNA levels of *GAPDH*. Data are mean±S.E.M of n=3 independent experiments; **F)** (left) Representative western blots and (right) quantification of the expression of the mitochondrial proteins TOMM20 and VDAC in proliferating and senescent (2 days after 10Gy X-ray) Control and RhebN153T expressing MEFs. Data are mean±S.E.M. of n=3 independent western blots. Asterisks denote statistical significant *P*<0.05 using one-way ANOVA.

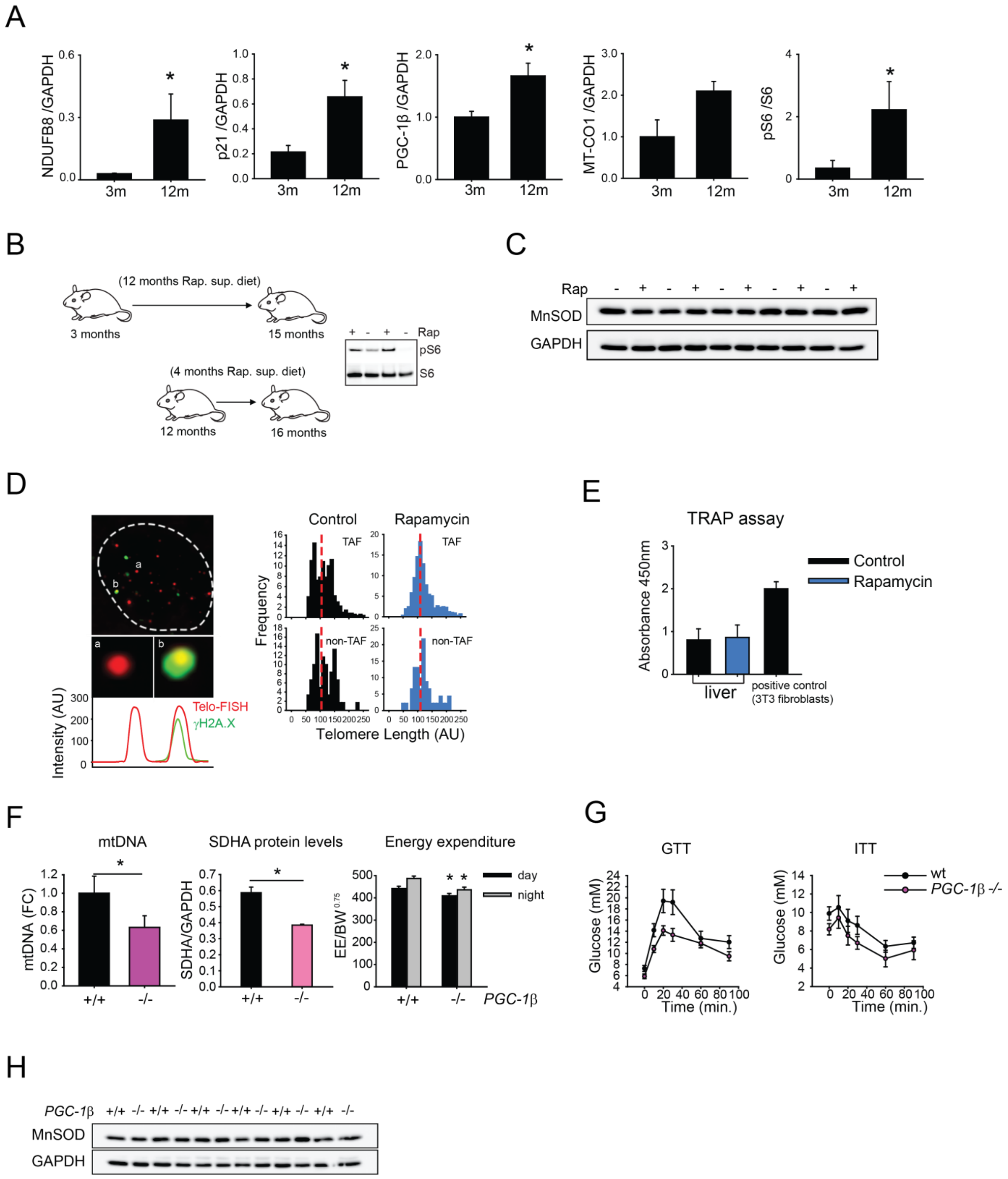

Appendix Figure S3 mTORC1-PGC-1β dependent increased mitochondrial content contributes to senescence *in vivo*. A) Quantification of NDUFB8, MT-CO1, pS6/S6, PGC-1β and p21 protein expression in liver tissue from wild-type C57BL/6 mice at 3 and 12 months of age. Data are mean±S.E.M n=3 mice per group; **B)** (left) Scheme of the experimental design of the mouse study involving rapamycin supplementation. Long-term rapamycin diet: mice at 3 months of age were fed with control or rapamycin diet for 12 months; Short -term rapamycin diet: mice at 12 months of age were fed with control or rapamycin diet for 4 months; (right) Representative western blot of the expression of pS6 and S6 in 16 months old mice liver with or without 4 months rapamycin supplementation; **C)** Western blots showing MnSOD expression on liver tissue from 15 months old mice fed with control or rapamycin diet for 12 months. Data are from n=5 mice per group; **D)** (left) Representative image of a hepatocyte stained for γH2A.X and telomere-FISH, from a 16

month old mouse fed with rapamycin for 4 months. Two telomeres of equal intensity are shown, one co-localising with γH2A.X and the other not; (right) Histograms showing telomere intensity for telomeres co-localizing (TAF) or not co-localising (non-TAF) with DNA damage foci in hepatocytes from 16 months old mice with or without 4 months rapamycin treatment. Data are from n=4 mice per group (intensity of 1000 telomeres was analysed per condition). Red dotted line represents median intensity. Mann-Whitney test shows no significant difference in the telomere intensities distribution between TAF and non-TAF; **E)** Telomere repeat amplification protocol (TRAP) assay in liver extracts (in comparison with positive control 3T3 mouse fibroblasts) from 16 months old mice with or without 4 months rapamycin treatment. Data are mean±S.E.M. of n=4 mice per group; **F)** (left) mtDNA copy number comparison at 7 months of age (data are mean±S.E.M n=4 mice per group); (middle) quantification of the mitochondrial protein SDHA by western blotting (data are mean±S.E.M n=5 mice per group) and (right) energy expenditure (EE) normalised per body weight (BW) at day and night periods in wild-type and *PGC-1β*$^{-/-}$ mice at 18 months of age. Data are measured as J/min and are mean±S.E.M n=9-10 mice per group; **G)** Glucose tolerance test (GTT) and Insulin tolerance test (ITT): kinetics of plasma glucose following intraperitoneal injection of 1 g/kg body mass of glucose in wild-type and *PGC-1β*$^{-/-}$ mice at 18 months of age. Data are mean±S.E.M n=9-10 mice per group; **H)** Western blots showing MnSOD expression on liver tissue from 18 months old wild type and *PGC-1β*$^{-/-}$ mice. Data are from n=6 mice per group. Asterisks denote statistical significance P<0.05 using two-tailed t-test and one-way ANOVA.

Appendix Table S1: Primer sequences for mtDNA copy number

Gene	Species		Sequence
B2M	human	Forward	5’ CCAGCAGAGAATGGAAAGTCAA
		Reverse	5’ TCTCTCTCCATTCTTCAGTAAGTCAACT
ND1	human	Forward	5’ CCCTAAAACCCGCCACATCT
		Reverse	5’ GAGCGATGGTGAGAGCTAAGGT
ND1	Mouse	Forward	5’ ACACTTATTACAACCCAAGAACACAT
		Reverse	5’ TCATATTATGGCTATGGGTCAGG
ND5	Mouse	Forward	5’ CCACGCATTCTTCAAAGCTA
		Reverse	5’ TCGGATGTCTTGTTCGTCTG

Appendix Table S2: Primer sequences for mRNA expression analysis

Gene	Species		Sequence
PGC-1β	Human	Forward	5’ AGTCAACGGCCTTGTGTTAAG
		Reverse	5’ ACAACTTCGGCTCTGAGACTG
PGC-1α	Human	Forward	5’ TGAGAGGGCCAAGCAAAG
		Reverse	5’ ATAAATCACACGGCGCTCTT
IL-6	Human	Forward	5’CAGGAGCCCAGCTATGAACT
		Reverse	5’GAAGGCAGCAGGCAACAC
IL-8	Human	Forward	5’GAGTGGACCACACTGCGCCA
		Reverse	5’TCCACAACCCTCTGCACCCAGT
COX5A	Human	Forward	5’CAAAGTGTAAACCGCATGGAT
		Reverse	5’TCCAGGTAACTGTTCACACTCAA
NDUFS8	Human	Forward	5’GTGCAGGACCTCCTGGTG
		Reverse	5’TCTCGGGATCCTGCATGT
ATP5G1	Human	Forward	5’ TGCAGGGTAGTAGGAGTGCAG
		Reverse	5’ TTAGACCCCTGGTACAACAGC
GAPDH	Human	Forward	5’AAATCCCATCACCATCTTCC
		Reverse	5’ GACTCCACGACGTACTCAGC
CXCL1	Mouse	Forward	5’ GACTCCAGCCACACTCCAAC
		Reverse	5’ TGACAGCGCAGCTCATTG
CXCL5	Mouse	Forward	5’ TGCGTTGTGTTTGCTTAACCG
		Reverse	5’ AGCTATGACTTCCACCGTAGG
Inhibin A	Mouse	Forward	5’ GATCATCACCTTTGCCGAGT
		Reverse	5’ TGGTCCTGGTTCTGTTAGCC
IL-6	Mouse	Forward	5’ CTACCAAACTGGATATAATCAGGA
		Reverse	5’ CCAGGTAGCTATGGTACTCCAGAA
p16	Mouse	Forward	5’ TTGCCCATCATCATCACCT
		Reverse	5’GGGTTTTCTTGGTGAAGTTCG
PGC-1β	Mouse	Forward	5’ CGCTCCAGGAGACTGAATCCAG
		Reverse	5’ CTTGACTACTGTCTGTGAGGC
β-actin	Mouse	Forward	5’ TAAGGCCAACCGTGAAAAG
		Reverse	5’ ACCAGAGGCATACAGGGACA

Appendix Table S3: Primary and secondary antibodies for Immunofluorescence

Primary antibody for	Species	Host	Dilution	Reference/Manufacturer
Ki67	Human Mouse	Rabbit polyclonal	1:250	ab15580 - Abcam
γ-H2A.X(Ser139)	Human	Mouse monoclonal	1:2000	05-636 - Millipore
γ-H2A.X(Ser139)	Human Mouse	Rabbit polyclonal	1:250	#9718 - Cell Signalling
53BP1	Human	Rabbit polyclonal	1:500	#4937 - Cell Signalling
53BP1	Human Mouse	Rabbit polyclonal	1:200	NB100-305 - Novus Biologicals
MT-CO1	Human Mouse	Mouse monoclonal	1:500	ab45918 - Abcam
SDHA	Human Mouse	Rabbit monoclonal	1:100	#11998 – Cell Signalling
BrdU		Mouse monoclonal	1:25	#347580 – BD Bioscieneces

Secondary antibodies	Dilution	Reference/Manufacturer
Anti-mouse Fluorescein-conjugated secondary antibody AlexaFluor 488	1:1000	A21042 - Molecular Probes
Anti-mouse Fluorescein-conjugated secondary antibody AlexaFluor 594	1:1000	A21044 - Molecular Probes
Anti-rabbit Fluorescein-conjugated secondary antibodyAlexaFluor 488	1:1000	A21212 - Molecular Probes
Anti-rabbit Fluorescein-conjugated secondary antibodyAlexaFluor 594	1:1000	A21213 - Molecular Probes
Anti-mouse Fluorescein-conjugated secondary antibodyAlexaFluor 647	1:1000	A21235 - Molecular Probes
Anti-rabbit Fluorescein-conjugated secondary antibodyAlexaFluor 647	1:1000	A21244 - Molecular Probes
**Anti-rabbit IgG Biotinylated (VECTASTAIN Elite ABC Kit (Rabbit IgG))**	1:200	PK-6101 – Vector Laboratories
**Fluorescein Avidin DCS**	1:500	A-2011- Vector Laboratories

Appendix Table S4: Primary and secondary antibodies for Western Blotting

Primary antibody for	Species	Host	Dilution	Reference/Manufacturer
γ-H2A.X(Ser139)	Human<br>Mouse	Rabbit<br>polyclonal	1:1000	#9718 - Cell Signalling
p21	Human	Rabbit<br>monoclonal	1:1000	#2947 - Cell signalling
PGC-1β	Human<br>Mouse	Rabbit<br>polyclonal	1:1000	ab61249 - Abcam
NDUFB8	Human<br>Mouse	Mouse<br>monoclonal	1:1000	ab110242 - Abcam
UQCRC2	Human<br>Mouse	Mouse<br>monoclonal	1:1000	ab14745 - Abcam
MT-CO1	Human<br>Mouse	Rabbit<br>monoclonal	1:250	ab14705 - Abcam
COXIV	Human<br>Mouse	Rabbit<br>polyclonal	1:1000	#4844 - Cell Signalling
SDHA	Human<br>Mouse	Mouse<br>monoclonal	1:1000	ab14715 - Abcam
TOMM20	Human<br>Mice	Mouse<br>monoclonal	1:1000	ab56783- Abcam
VDAC1/Porin	Human<br>Mouse	Mouse<br>monoclonal	1:1000	ab14734 - Abcam
S6	Human<br>Mouse	Rabbit<br>monoclonal	1:1000	#2217- Cell signalling
S6(Ser235/236)	Human<br>Mouse	Rabbit<br>monoclonal	1:1000	#4858- Cell signalling
p70S6K	Human<br>Mouse	Rabbit<br>polyclonal	1:1000	#9202 - Cell signalling
p70S6K(Thr389)	Human<br>Mouse	Rabbit<br>polyclonal	1:1000	#9205 - Cell signalling
Akt	Human<br>Mouse	Rabbit<br>polyclonal	1:1000	#9272 - Cell signalling
p-Akt(S473)	Human<br>Mouse	Rabbit<br>polyclonal	1:1000	#9271 - Cell signalling
β-tubulin	Human<br>Mouse	Rabbit<br>polyclonal	1:2000	#2146 - Cell signalling
α-tubulin	Human<br>Mouse	Mouse<br>monoclonal	1:2000	T9026 - Sigma Aldrich
GAPDH	Human<br>Mouse	Rabbit<br>monoclonal	1:5000	#5174 - Cell signalling

FLAG	-	Mouse	1:1000	**F3165 -** Sigma Aldrich
FLAG	-	Rabbit	1:1000	**F7425 -** Sigma Aldrich
**Secondary antibodies**			**Dilution**	**Reference/Manufacturer**
Goat anti-rabbit IgG -HRP conjugated			1:5000	A0545 - Sigma-Aldrich
Rabbit anti-mouse IgG -HRP conjugated			1:5000	A2554 - Sigma-Aldrich
