# Supplementary material for: Mitochondria are required for pro‐ageing features of the senescent phenotype
Source: EMBO J. 2016 Feb 4;35(7):724–42. doi: 10.15252/embj.201592862 (PMC4818766; doi:10.15252/embj.201592862)
Supplement: Supplementary file 2 — Expanded View Figures PDF [file EMBJ-35-724-s002.pdf]

Expanded View Figures

Figure EV1. Mitochondria are key factors for the development of pro-ageing features of cellular senescence.

A Effects of CCCP treatment on mitochondrial depletion, immediately (left) and 16 days (right) after 48 h of 12.5 μM CCCP treatment on irradiated (IR) MRC5 fibroblasts. Cells were irradiated with 20-Gy X-ray and treated with 12.5 μM CCCP at day 2 after irradiation. After 48 h of CCCP treatment, cells were either immediately collected for analysis (4 days after IR) or were kept in culture with normal serum-supplemented medium for 16 days and then harvested for analysis (20 days after IR). Western blots are representative of three independent experiments.

B T.E.M. images of senescent Parkin-expressing MRC5 fibroblasts at 10 and 20 days after irradiation, pre-treated or not with CCCP. Scale bar = 1 μm; red arrows denote mitochondria. Note: when conducting 3D-EM of 20 days after IR, one cell appeared to have lost Parkin and did not show the depletion of mitochondria (indistinguishable from controls), while those where depletion was complete (the majority) were devoid of intact mitochondria.

C Levels of secreted IL-6 and IL-8 proteins (measured by ELISA) in proliferating and senescent (10 days after 20-Gy X-ray) control and Parkin-expressing MRC5 fibroblasts with or without 12.5 μM CCCP treatment. Data are representative of two independent experiments.

D (left) Representative flow cytometry histogram of mitochondrial mass staining (NAO) in parental and rho0 143B osteosarcoma cells (data are representative of three independent experiments), (middle) quantification of ROS levels (DHE intensity) and (right) mRNA abundance of the SASP factor IL-6 in parental and rho0 cells following 10-Gy X-ray. Data are mean ± SEM of n = 3 independent experiments; asterisks denote a significance by one-way ANOVA at $P < 0.05$.

E Representative images of the proliferation marker Ki67 (representative of two independent experiments), quantification of population doublings (PD) and BrdU-positive cells of proliferating and senescent (10 and/or 20 days after 20-Gy X-ray) control and Parkin-expressing MRC5 fibroblasts with or without 12.5 μM CCCP treatment. Data are mean ± SEM of n = 3 independent experiments for PD analysis and mean ± SD of 10 random panes for BrdU analysis.

F Representative Western blots of mTORC1 activity, measured by p70S6K phosphorylation (T389), in senescent (10 days after 20-Gy X-ray) control (C) and Parkin-expressing (P) MRC5 fibroblasts. Data are representative of 3 independent experiments.

G Representative Western blots showing the absence of mitochondrial proteins (NDUFB8, SDHA, UQCRC2 and TOMM20), expression of p21 and mTOR activity (measured by phosphorylation of the p70S6K (T389)) in proliferating control and Parkin-expressing MRC5 fibroblasts (10 days after 48 h of 12.5 μM CCCP treatment). Data are representative of two independent experiments.

H Quantification of BrdU-positive cells (data are mean ± SD of n = 2 independent experiments), Sen-β-Gal activity (data are mean ± SD from 10 random planes), IL-6 secretion measured by ELISA (data are representative of two independent experiments) and ROS levels measured by DHE intensity (data are mean ± SD of n = 3 technical repeats) of proliferating control and Parkin-expressing MRC5 fibroblasts (after 48 h of 12.5 μM CCCP treatment).

I Steady-state cellular ATP levels were measured using an ATP luciferase Kit (Invitrogen) in proliferating control and Parkin-expressing MRC5 fibroblasts (after 48 h of 12.5 μM CCCP treatment). Data are mean ± SEM of n = 3 independent experiments.

© 2016 The Authors

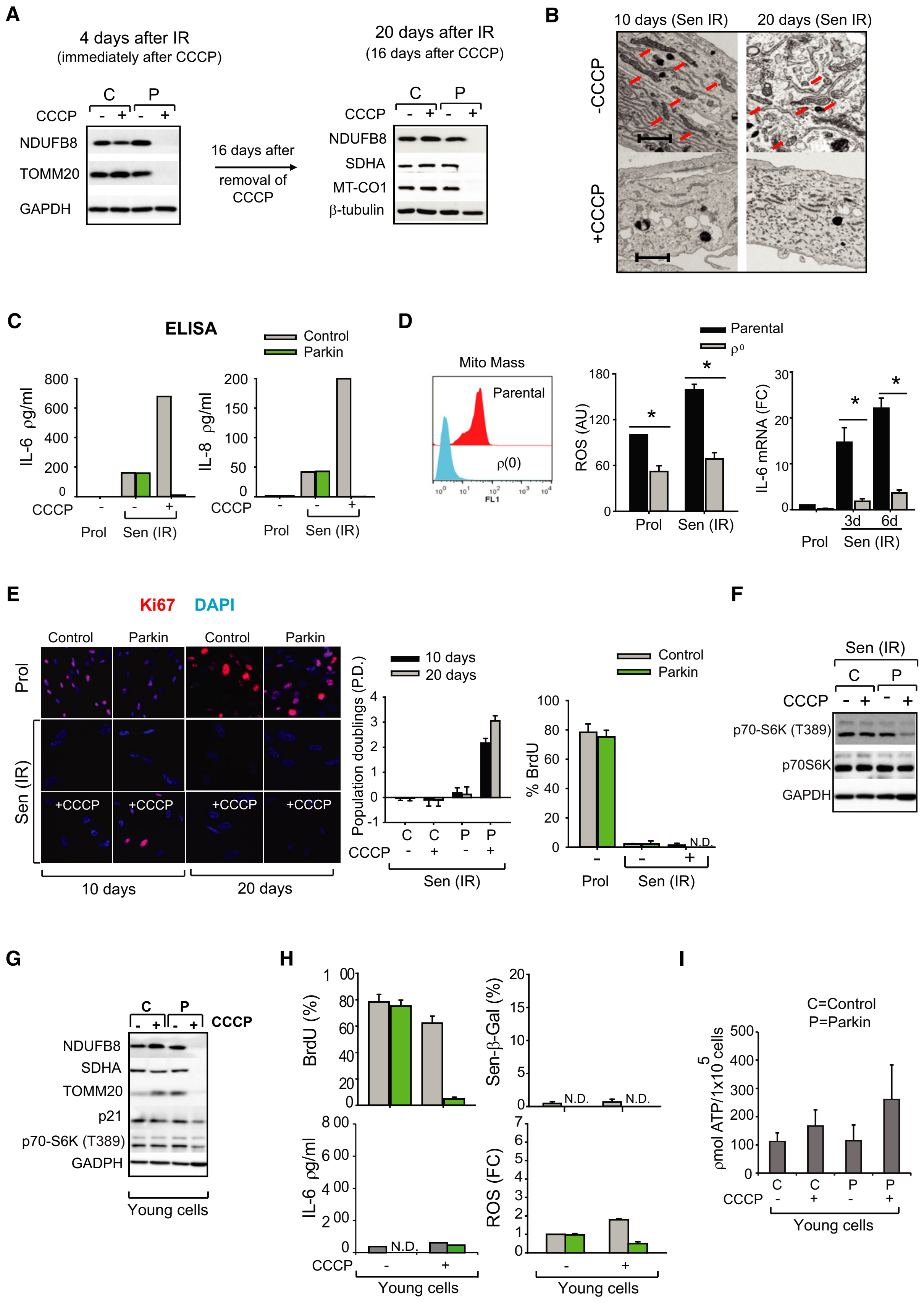
A
4 days after IR
(immediately after CCCP)
C P
CCCP - + - +
NDUFB8
TOMM20
GAPDH
16 days after removal of CCCP
20 days after IR
(16 days after CCCP)
NDUFB8
SDHA
MT-CO1
β-tubulin
B
10 days (Sen IR)
20 days (Sen IR)
-CCCP
+CCCP
C
ELISA
Control
Parkin
IL-6 ρg/ml
IL-8 ρg/ml
Prol
Sen (IR)
D
Mito Mass
Parental
ρ(0)
FL1
ROS (AU)
IL-6 mRNA (FC)
3d
6d
E
Ki67
DAPI
Control
Parkin
Prol
Sen (IR)
+CCCP
10 days
20 days
Population doublings (P.D.)
% BrdU
N.D.
F
Sen (IR)
p70-S6K (T389)
p70S6K
GAPDH
G
p21
GADPH
Young cells
H
BrdU (%)
Sen-β-Gal (%)
IL-6 ρg/ml
ROS (FC)
I
C=Control
P=Parkin
ρmol ATP/1x10^5 cells

Figure EV1.

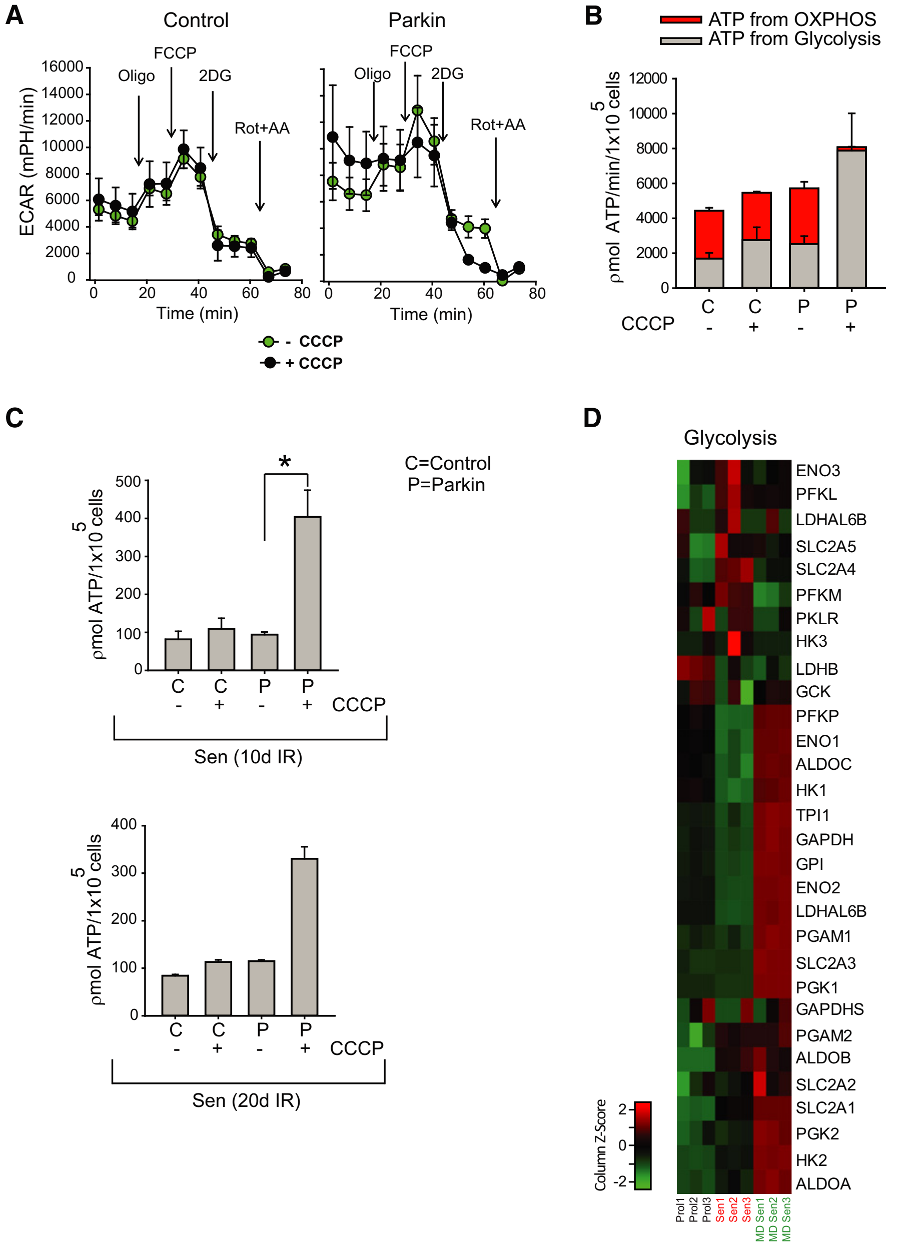

Figure EV2. Alleviation of the senescent phenotype in mitochondria-depleted cells does not compromise ATP generation.

A Extracellular acidification rate (ECAR) of senescent (10 days after 20 Gy) control and Parkin-expressing MRC5 fibroblasts with or without 12.5 μM CCCP treatment. ECAR was measured using a Seahorse XF24 analyzer. Data are representative graphs from the Seahorse XF24 analyzer software. Data are mean ± SD n = 4 technical repeats (representative of two independent experiments).

B ATP production rate in senescent (10 days after 20 Gy) control (C) and Parkin-expressing (P) MRC5 fibroblasts with or without 12.5 μM CCCP treatment (calculated from the OCR and ECAR measured using a Seahorse XF24 analyzer). ATP production by mitochondria was calculated by multiplying the ATP turnover ((basal OCR) – (OCR with oligomycin) by the established phosphorus/oxygen (P/O) ratio of 2.3 (Brand, 2005). ATP production by glycolysis is considered to have a 1:1 ratio with lactate production. The extracellular acidification rate is mainly due to lactate and bicarbonate production and, when calibrated as the proton production rate, indicates glycolytic rate (Birket *et al* 2011; Wu *et al*, 2007). Data are mean ± SD, n = 4 technical repeats (representative of two independent experiments).

C Steady-state cellular ATP levels were measured using an ATP luciferase assay (Invitrogen) in senescent control and Parkin-expressing MRC5 fibroblasts with or without 12.5 μM CCCP treatment, at 10 days (data are mean ± SEM, n = 3 independent experiments) and 20 days (data are mean ± SD from n = 3 technical repeats) after 20-Gy X-ray. Asterisk denotes a statistical significance at $P < 0.05$ using one-way ANOVA.

D Heatmap of the RNA-seq analysis of glycolysis-associated gene expression in proliferating and senescent (10 days after 20 Gy) Parkin-expressing MRC5 fibroblasts with or without 12.5 μM CCCP treatment. Red indicates up-regulated genes; green indicates down-regulated genes. Data are from n = 3 independent experiments.

 © 2016 The Authors

Figure EV3. Mitochondria are important for the development of pro-ageing features of oxidative stress-induced, oncogene-induced and replicative senescence.

A (left) Scheme showing experimental design: control and Parkin-expressing MRC5 fibroblasts were induced to senescence with 400 μM of H_2O_2 for 1 h in serum-free medium. Two days upon the induction of senescence with H_2O_2, cells were treated with 12.5 μM CCCP for 48 h and then cultured in normal serum-supplemented medium during 6 or 16 days for senescence markers analysis (10 and 20 days following the induction of senescence, respectively); (right) Representative images of the mitochondrial protein SDHA (immunofluorescence) in senescent control and Parkin-expressing MRC5 fibroblasts, 10 days after the induction of senescence with 400 μM H_2O_2. Scale bar = 10 μm.

B Quantification of Sen-β-Gal- (data are mean ± SD of n = two independent experiments) and BrdU-positive (data are mean ± SD of 10 random planes) proliferating and senescent (10 and 20 days after the induction of senescence with 400 μM H_2O_2) control and Parkin-expressing MRC5 fibroblasts with or without 12.5 μM CCCP treatment.

C Levels of secreted IL-8 and IL-6 proteins (measured by ELISA) in proliferating and senescent (10 days after the induction of senescence with 400 μM H_2O_2) control and Parkin-expressing MRC5 fibroblasts with or without 12.5 μM CCCP treatment. Data are representative of two independent experiments.

D (left) Scheme showing experimental design: oncogene-induced senescence (OIS) experiments were performed by treating Parkin-expressing ER-RAS/IMR-90 fibroblasts with 12.5 μM CCCP for 48 h, followed by ER-RAS induction with 100 nM of 4-hydroxytamoxifen (4-OHT). Cells were harvested 7 days after ER-RAS induction; (right) Representative Western blots showing the expression of the mitochondrial proteins NDUFB8, SDHA, UQCRC2 and COXIV in OIS Parkin-expressing ER-RAS/IMR-90 fibroblasts. Data are representative of two independent experiments.

E Representative Western blots showing the expression of p21 and p16 proteins in OIS Parkin-expressing ER-RAS/IMR-90 fibroblasts, 7 days after ER-RAS induction. Data are representative of two independent experiments.

F Quantification of Sen-β-Gal- and EdU-positive proliferating and OIS Parkin-expressing ER-RAS/IMR-90 fibroblasts, 7 days after ER-RAS induction. Data are mean ± SD of n = 2 independent experiments.

G Scheme illustrating the experimental design: Parkin-expressing MRC5 fibroblasts were cultured until replicative senescence (RS). RS cells were treated with 12.5 μM CCCP for 48 h (until day 2) and then cultured in normal serum-supplemented medium for 6 days.

H Representative Western blots showing the absence of mitochondrial proteins from the different mitochondrial complexes: NDUFB8 (complex I), SDHA (complex II), UQCRC2 (complex III) and MTCO-1 (complex IV) and expression of p21 and p16 in replicative senescent Parkin-expressing MRC5 fibroblasts, 6 days after 12.5 μM CCCP treatment. Data are representative of two independent experiments.

I ROS analysis (DHE intensity) in replicative senescent Parkin-expressing MRC5 fibroblasts, 6 days after 12.5 μM CCCP treatment. Data are mean ± SD of n = 3 technical repeats and are representative of two independent experiments.

J Quantification of Sen-β-Gal- and BrdU-positive cells in proliferating and replicative senescent (RS) Parkin-expressing MRC5 fibroblasts, 6 days after 12.5 μM CCCP treatment. Data are mean ± SD of 10 random planes and are representative of two independent experiments.

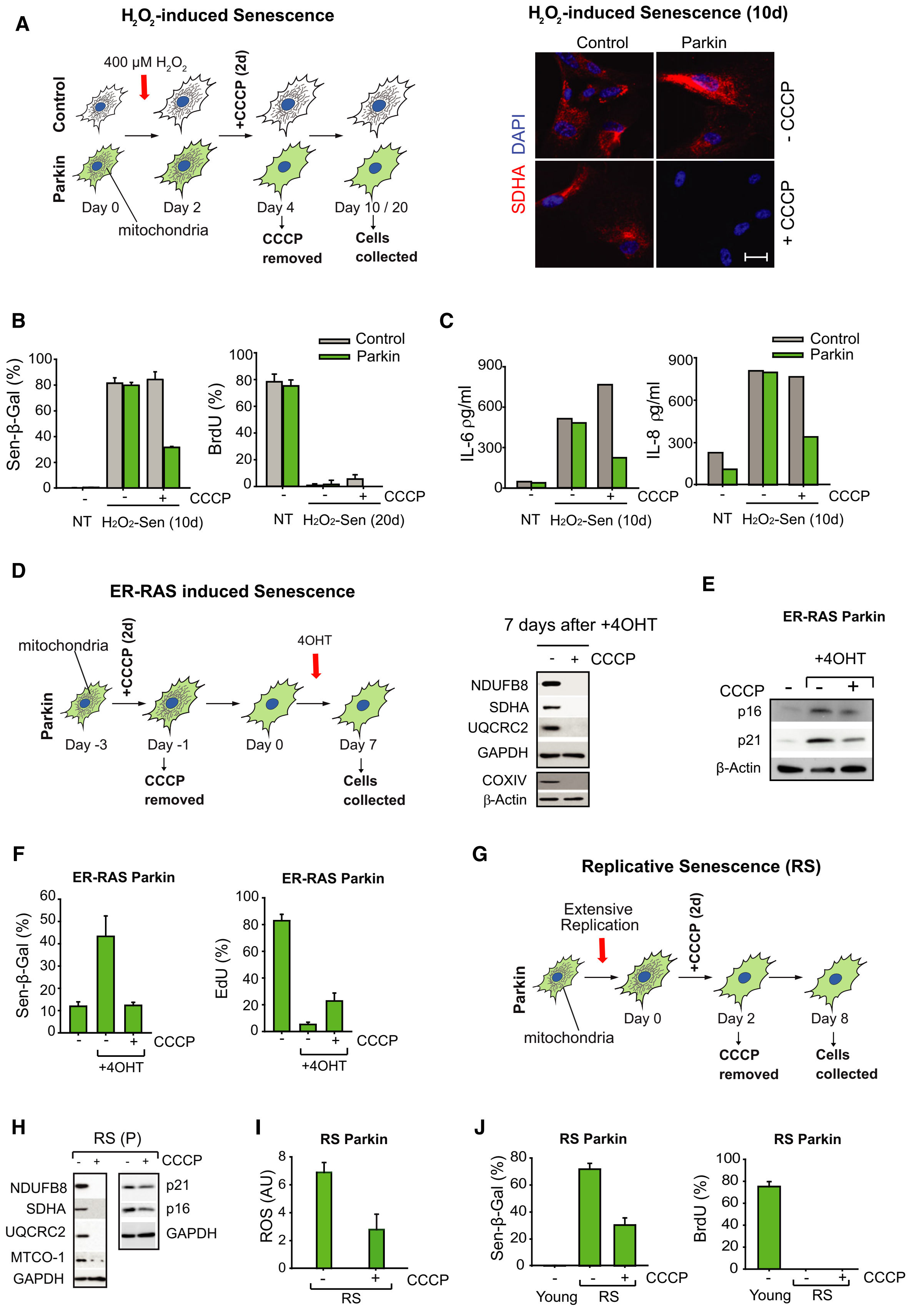

Figure EV3.

 © 2016 The Authors

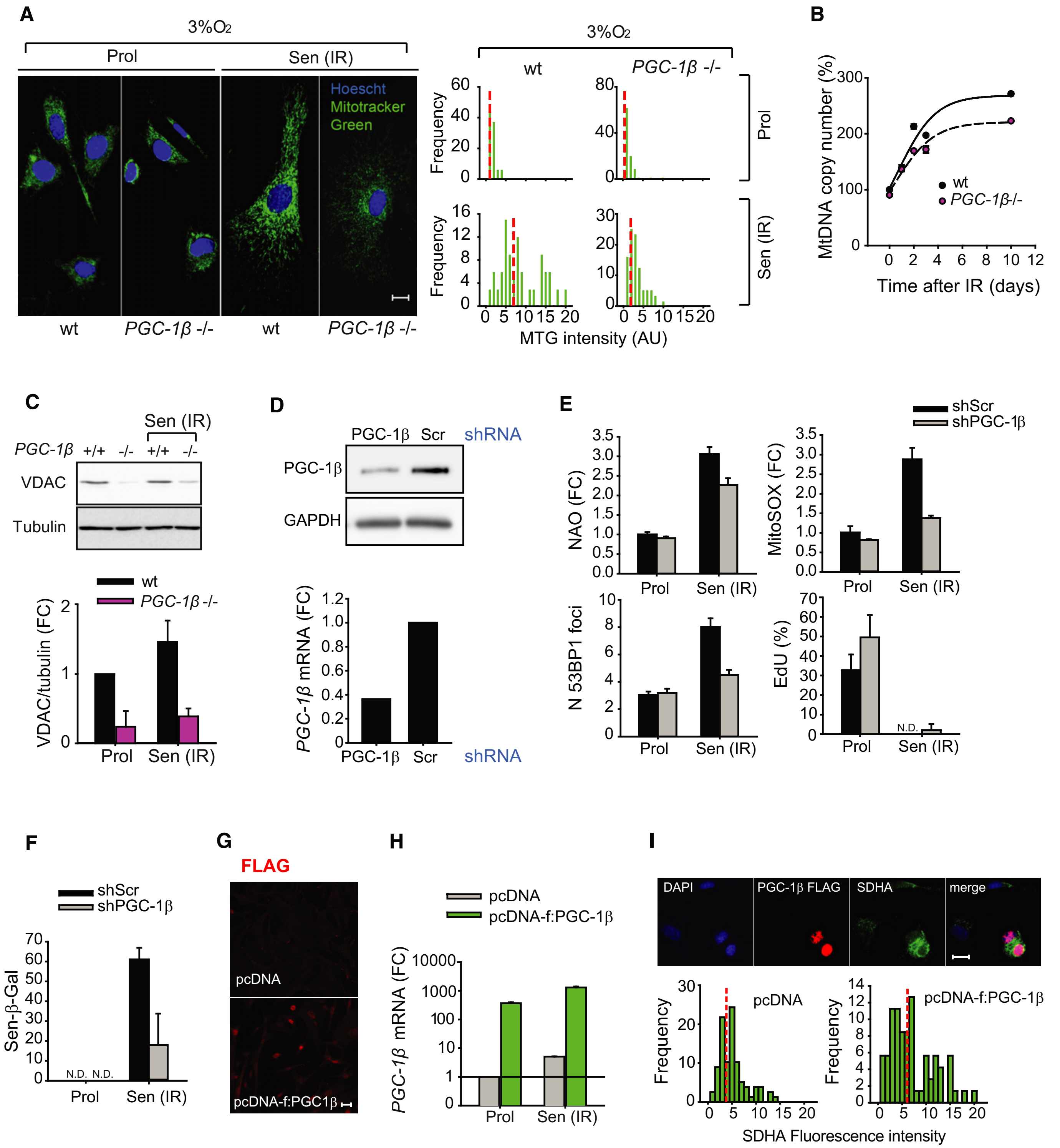
A
3%O2
Prol
Sen (IR)
Hoescht
Mitotracker
Green
wt
PGC-1β -/-
wt
PGC-1β -/-
3%O2
wt
PGC-1β -/-
Prol
Sen (IR)
Frequency
MTG intensity (AU)
B
MtDNA copy number (%)
wt
PGC-1β-/-
Time after IR (days)
C
Sen (IR)
PGC-1β +/+ -/- +/+ -/-
VDAC
Tubulin
VDAC/tubulin (FC)
Prol
D
PGC-1β
Scr
shRNA
GAPDH
PGC-1β mRNA (FC)
E
shScr
shPGC-1β
NAO (FC)
MitoSOX (FC)
N 53BP1 foci
EdU (%)
N.D.
F
Sen-β-Gal
N.D. N.D.
G
FLAG
pcDNA
pcDNA-f:PGC1β
H
pcDNA
pcDNA-f:PGC-1β
I
DAPI
PGC-1β FLAG
SDHA
merge
SDHA Fluorescence intensity

Figure EV4.

Figure EV4. PGC-1β-dependent mitochondrial biogenesis modulates cellular senescence.

A (left) Representative images and (right) histograms showing the distribution of MitoTracker green (MTG) immunofluorescence in proliferating and senescent (3 days after 10-Gy X-ray) wild-type and *PGC-1β*$^{-/-}$ MEFs. Scale bar = 10 μm. Dashed red lines indicate median MitoTracker green intensity (50–100 cells were quantified per condition).

B Kinetics of mtDNA copy number after 10-Gy X-ray in wild-type and *PGC-1β*$^{-/-}$ MEFs. Data are mean ± SEM of n = 3 independent experiments.

C Expression of the mitochondrial outer membrane protein VDAC in wild-type and *PGC-1β*$^{-/-}$ MEFs 1 day after 10-Gy X-ray. Data are mean ± SEM of n = 3 independent experiments.

D Knockdown efficiency of PGC-1β in human MRC5 fibroblasts infected with a control vector (shScr) and with a shPGC-1β vector by Western blot and qPCR.

E Quantification of mitochondrial mass (NAO intensity), mitochondrial ROS (MitoSOX intensity), mean number (N) of 53PB1 foci and Edu-positive cells in proliferating and senescent MRC5 fibroblasts infected with a control (shScr) and shPGC-1β vector. Mitochondrial mass and ROS measurements were performed 3 days after 20-Gy X-ray (data are mean ± SD of n = 3 technical replicates). 53BP1 foci and EdU incorporation were analysed 10 days after 20-Gy X-ray (data are mean ± SD of 80–100 cells/condition for 53BP1 analysis and 10 random planes for the EdU incorporation analysis).

F Quantification of Sen-β-Gal-positive cells in proliferating and senescent (10 days after 20-Gy X-ray) MRC5 fibroblasts infected with control (shScr) and shPGC-1β vectors. Data are mean ± SD 10 random planes.

G Representative image of FLAG-PGC-1β immunostaining 2 days following the transfection of a pcDNA empty vector and pcDNAf:PGC-1β in wild-type MEFs. Scale bar = 20 μm.

H mRNA expression of *PGC-1β* in proliferating and senescent (10 days after 10-Gy X-ray) wild-type MEFs following the transfection with pcDNA empty vector and pcDNAf:PGC-1β. Data are mean ± SEM of n = 3 independent experiments.

I (top) Representative image of FLAG-PGC-1β (red) and the mitochondrial protein SDHA (green) double immunostaining and (bottom) histograms showing the distribution of SDHA fluorescence, 2 days after transfection with a pcDNA empty vector and pcDNAf:PGC-1β in wild-type MEFs. Scale bar = 10 μm. Dashed red lines indicate median SDHA fluorescence (100 cells were quantified per condition).

© 2016 The Authors

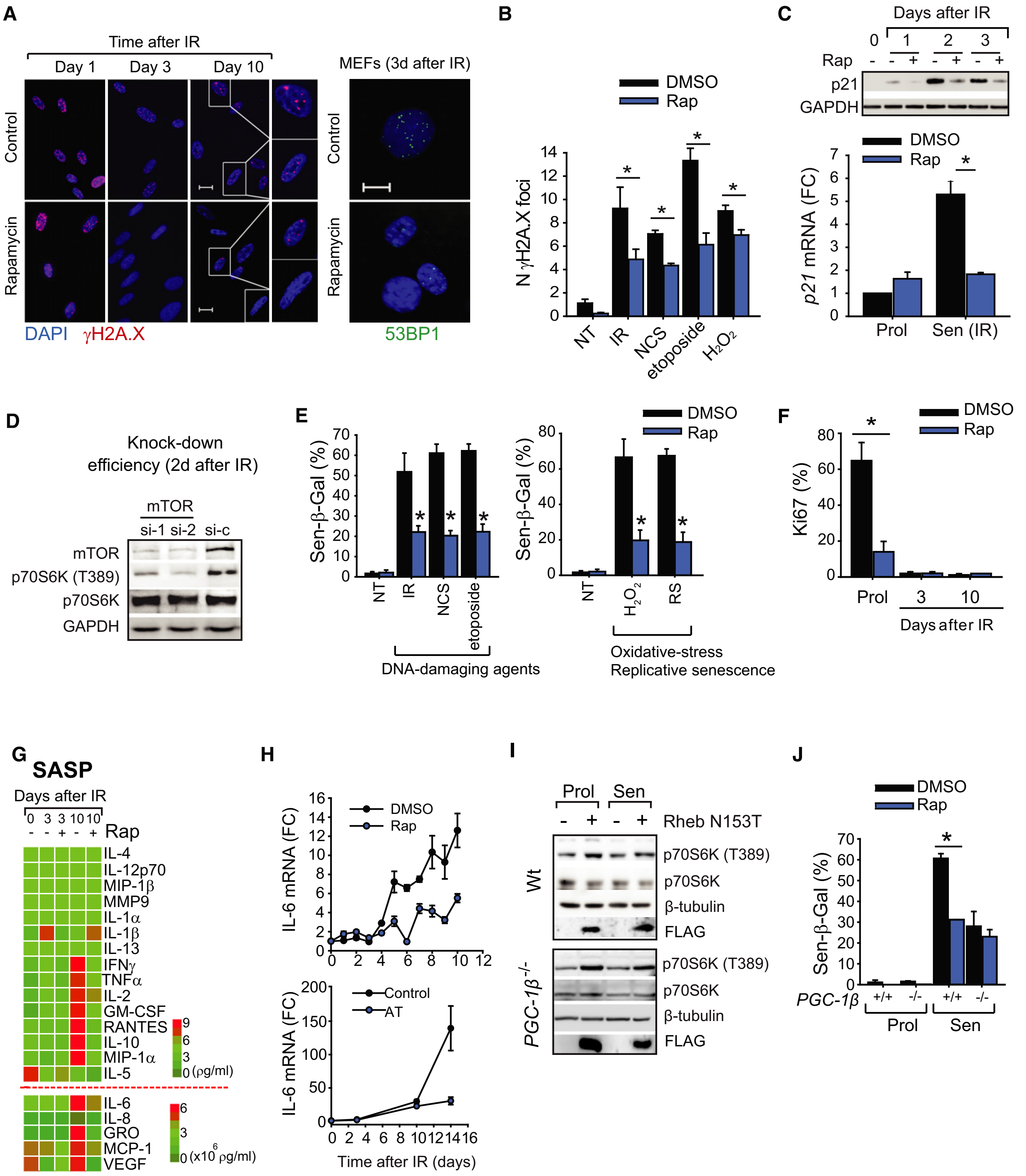
A
Time after IR
Day 1
Day 3
Day 10
MEFs (3d after IR)
Control
Rapamycin
DAPI γH2A.X
53BP1
B
DMSO
Rap
N γH2A.X foci
NT
IR
NCS
etoposide
H2O2
C
Days after IR
Rap
p21
GAPDH
p21 mRNA (FC)
Prol
Sen (IR)
D
Knock-down efficiency (2d after IR)
mTOR
si-1 si-2 si-c
mTOR
p70S6K (T389)
p70S6K
GAPDH
E
Sen-β-Gal (%)
DNA-damaging agents
Oxidative-stress
Replicative senescence
RS
F
Ki67 (%)
Days after IR
G
SASP
Days after IR
IL-4
IL-12p70
MIP-1β
MMP9
IL-1α
IL-1β
IL-13
IFNγ
TNFα
IL-2
GM-CSF
RANTES
IL-10
MIP-1α
IL-5
(ρg/ml)
IL-6
IL-8
GRO
MCP-1
VEGF
H
IL-6 mRNA (FC)
Control
AT
Time after IR (days)
I
Prol
Sen
Rheb N153T
Wt
PGC-1β-/-
p70S6K (T389)
p70S6K
β-tubulin
FLAG
J
PGC-1β
+/+
-/-

Figure EV5.

Figure EV5. mTORC1 activation contributes to the senescent phenotype via PGC1-β-dependent mitochondrial biogenesis and ROS-mediated activation of a DDR.

A (left) Representative images of γH2A.X (red foci) immunofluorescence in MRC5 fibroblasts at different time points after 20-Gy X-ray with or without 100 nM rapamycin supplementation (scale bar = 5 μm) and (right) representative images of 53BP1 (green foci) immunofluorescence in MEFs, 3 days after 10-Gy X-ray with or without 100 nM rapamycin supplementation (scale bar = 5 μm).

B Effect of genomic damage- or oxidative stress-induced senescence on the mean number (N) of γH2A.X foci in MRC5 fibroblasts with or without 100 nM rapamycin. Cells were analysed 3 days after the induction of senescence and treatment with rapamycin. Data are mean ± SEM of n = 3 independent experiments; asterisks denote a statistical significance at $P < 0.05$ using one-way ANOVA.

C (top) Representative Western blot showing the inhibition of p21 protein expression with 100 nM rapamycin at different time points (days) after 20-Gy X-ray in MRC5 fibroblasts. Data are representative of three independent experiments; (bottom) *p21* mRNA levels at 3 days after 20-Gy X-ray with or without 100 nM rapamycin treatment in MRC5 fibroblasts. Data are mean ± SEM of n = 3 independent experiments; asterisks denote a statistical significant at $P < 0.05$ using one-way ANOVA.

D Western blots showing the knockdown efficiency of two different siRNAs against mTOR and its effects on p70S6K phosphorylation (T389), 2 days after 20-Gy X-ray in MRC5 fibroblasts. Data are representative of two independent experiments.

E Effect of 100 nM rapamycin supplementation on Sen-β-Gal activity in MRC5 fibroblasts induced to senescence following treatment with 20-Gy X-ray irradiation, 80 ng ml^{-1} neocarzinostatin (NCS), 50 μM etoposide, 400 μM H_2O_2 and replicative exhaustion (RS). Cells were analysed 10 days after the induction of senescence and treatment with rapamycin. Data are mean ± SEM of n = 3 independent experiments (at least 80 cells were analysed per condition). Asterisks denote a statistical significance at $P < 0.05$ using one-way ANOVA.

F Quantification of Ki67-positive MRC5 fibroblasts 3 and 10 days following 20-Gy X-ray irradiation with or without 100 nM rapamycin treatment. Data are mean ± SEM of n = 4 independent experiments; asterisks denote a statistical significance at $P < 0.05$ using one-way ANOVA.

G Secreted protein array of a variety of inflammatory proteins following 20-Gy X-ray-induced senescence in MRC5 fibroblasts with or without 100 nM rapamycin treatment (3 and 10 days after 20-Gy X-ray). Data are mean of three independent experiments.

H mRNA expression of IL-6 after 20-Gy X-ray (left) with or without 100 nM rapamycin treatment in MRC5 fibroblasts and (right) in human fibroblasts from an AT patient. Data are mean ± SEM of n = 3 independent experiments (for MRC5+Rap cells) and mean ± SD of n = 2 independent experiments (for AT cells).

I Representative Western blots of mTORC1 activity, measured by p70S6K phosphorylation (T389) in wild-type and *PGC-1β*$^{-/-}$ MEFs overexpressing RhebN153T (1 day after 10-Gy irradiation). Data are representative of three independent experiments.

J Effect of rapamycin supplementation on Sen-β-Gal activity in wild-type and *PGC-1β*$^{-/-}$ MEFs, 10 days after 10-Gy X-ray. Data are mean ± SEM of n = 3 independent experiments (at least 100 cells were analysed per condition). Asterisks denote a statistical significance at $P < 0.05$ using one-way ANOVA.

 © 2016 The Authors
