# Supplementary material for: Mitochondria are required for pro‐ageing features of the senescent phenotype
Source: EMBO J. 2016 Feb 4;35(7):724–42. doi: 10.15252/embj.201592862 (PMC4818766; doi:10.15252/embj.201592862)
Supplement: Supplementary file 3 — Movie EV1 [file EMBJ-35-724-s003.zip › EMBOJ_92862_Movie_EV1/Movie_1_Figure_Legend.rtf]

3D EM of Senescent MRC5 Parkin fibroblasts (20 days after 20Gy).Stack of 201 20nm sections of senescent MRC5 fibroblasts expressing Parkin (20days after IR) covering a depth of 4um
