# Supplementary material for: Mitochondria are required for pro‐ageing features of the senescent phenotype
Source: EMBO J. 2016 Feb 4;35(7):724–42. doi: 10.15252/embj.201592862 (PMC4818766; doi:10.15252/embj.201592862)
Supplement: Supplementary file 4 — Movie EV2 [file EMBJ-35-724-s004.zip › EMBOJ_92862_Movie_EV2/Movie_2_Figure_Legend.rtf]

3D EM of Senescent MRC5 Parkin fibroblasts pre-treated with CCCP (20 days after 20Gy).  Stack of 291 20nm sections of senescent MRC5 fibroblasts expressing Parkin pre-treated with CCCP (20days after IR) covering a depth of 6um.
