# Supplementary material for: Mitochondria are required for pro‐ageing features of the senescent phenotype
Source: EMBO J. 2016 Feb 4;35(7):724–42. doi: 10.15252/embj.201592862 (PMC4818766; doi:10.15252/embj.201592862)
Supplement: Supplementary file 5 — Movie EV3 [file EMBJ-35-724-s005.zip › EMBOJ_92862_Movie_EV3/Movie_3_Figure_Legend.rtf]

Live cell imaging of MRC5 Parkin fibroblasts after induction of senescence by IR.MRC5 fibroblasts expressing Parkin were irradiated with 20Gy and imaged starting at 6-12 days after.
