# Supplementary material for: Mitochondria are required for pro‐ageing features of the senescent phenotype
Source: EMBO J. 2016 Feb 4;35(7):724–42. doi: 10.15252/embj.201592862 (PMC4818766; doi:10.15252/embj.201592862)
Supplement: Supplementary file 6 — Movie EV4 [file EMBJ-35-724-s006.zip › EMBOJ_92862_Movie_EV4/Movie_4_Figure_Legend.rtf]

Live cell imaging of MRC5 Parkin fibroblasts pre-treated with CCCP after induction of senescence by IRMRC5 fibroblasts expressing Parkin were irradiated with 20Gy, treated for 2 days with CCCP and imaged starting at 5-12 days after. Western blotting confirmed absence of mitochondrial proteins.
